# Inhibition of MZF1/c-MYC Axis by Cantharidin Impairs Cell Proliferation in Glioblastoma

**DOI:** 10.3390/ijms232314727

**Published:** 2022-11-25

**Authors:** Chie-Hong Wang, Hsuan-Cheng Wu, Chen-Wei Hsu, Yun-Wei Chang, Chiung-Yuan Ko, Tsung-I Hsu, Jian-Ying Chuang, Tsui-Hwa Tseng, Shao-Ming Wang

**Affiliations:** 1 Neuroscience and Brain Disease Center, China Medical University, Taichung 404333, Taiwan; 2 Department of Neurology, China Medical University Hospital, Taichung 404327, Taiwan; 3 Cell Therapy Center, China Medical University Hospital, Taichung 404327, Taiwan; 4 Ph.D. Program in Medical Neuroscience, College of Medical Science and Technology, Taipei Medical University, Taipei 110301, Taiwan; 5 International Master Program in Medical Neuroscience, College of Medical Science and Technology, Taipei Medical University, Taipei 110301, Taiwan; 6 Department of Medical Applied Chemistry, Chung Shan Medical University, Taichung 402306, Taiwan; 7 Depatment of Medical Education, Chung Shan Medical University Hospital, Taichung 402306, Taiwan; 8 Graduate Institute of Biomedical Sciences, China Medical University, Taichung 404333, Taiwan

**Keywords:** Myeloid zinc finger 1, glioblastoma multiforme, cell proliferation, cantharidin and norcantharidin

## Abstract

Myeloid zinc finger 1 (MZF1), also known as zinc finger protein 42, is a zinc finger transcription factor, belonging to the Krüppel-like family that has been implicated in several types of malignancies, including glioblastoma multiforme (GBM). MZF1 is reportedly an oncogenic gene that promotes tumor progression. Moreover, higher expression of MZF1 has been associated with a worse overall survival rate among patients with GBM. Thus, MZF1 may be a promising target for therapeutic interventions. Cantharidin (CTD) has been traditionally used in Chinese medicine to induce apoptosis and inhibit cancer cell proliferation; however, the mechanism by which CTD inhibits cell proliferation remains unclear. In this study, we found that the expression of MZF1 was higher in GBM tissues than in adjacent normal tissues and low-grade gliomas. Additionally, the patient-derived GBM cells and GBM cell lines presented higher levels of MZF1 than normal human astrocytes. We demonstrated that CTD had greater anti-proliferative effects on GBM than a derivative of CTD, norcantharidin (NCTD). MZF1 expression was strongly suppressed by CTD treatment. Furthermore, MZF1 enhanced the proliferation of GBM cells and upregulated the expression of c-MYC, whereas these effects were reversed by CTD treatment. The results of our study suggest that CTD may be a promising therapeutic agent for patients with GBM and suggest a promising direction for further investigation.

## 1. Introduction

Glioblastoma multiforme (GBM) is the deadliest and most malignant form of brain tumor, comprising approximately 57% of all gliomas [1]. After the first diagnosis and treatment (e.g., surgery, radiotherapy, and chemotherapy) [2], patients with GBM usually survive within 12 to 14 months, resulting in major personal and family issues as well as a heavy economic burden at the societal level. GBM is believed to be formed from stem cells or progenitor cells, especially glioma stem cells and oligodendrocyte precursor cells [3]. Currently, GBM has no effective treatment owing to abnormal cell proliferation, drug resistance, and glioma stem cell formation [4,5,6]. Because of the importance of abnormal proliferative behavior in GBM, further investigation is needed into the underlying molecular regulation and mechanisms for cancer initiation, progression, and resistance.

Among the Krüppel family of proteins, MZF1 influences gene transcription and contributes to cancer progression [7,8,9]. MZF1 reportedly interacts with c-MYC to promote cancer progression in colorectal carcinoma [8], lung adenocarcinoma [9], and glioma [4]. MZF1 is also capable of promoting breast cancer invasion and metastasis by increasing the expression of cysteine cathepsins B and L in lysosomes [7]. MZF1 expression is known to play a significant role in cancer progression [10,11]; however, only little is known about how MZF1 can be inhibited.

Cantharidin (CTD), a terpene compound, is the principal bioactive component of *Mylabris* [12,13]. Further, CTD was identified as a protein phosphatase 2A inhibitor [14]. In addition to inhibiting osteosarcoma proliferation and metastasis [11], CTD induces apoptosis and differentiation in acute myeloid leukemia [15] and reduces hepatocellular carcinoma development [16]. CTD demonstrates anti-cancer effects, such as reducing osteosarcoma proliferation and metastasis, inducing acute myeloid leukemia apoptosis and differentiation, and reducing hepatocellular carcinoma development. Furthermore, norcantharidin (NCTD) is a demethylated form of CTD that also prevents cancer progression by accelerating cell apoptosis [17] and suppressing cell growth [10]; however, the underlying mechanism of CTD and NCTD on inhibiting glioblastoma proliferation is still needed to be dissected.

We observed higher expression of MZF1 in glioma cells compared with normal human astrocytes, and MZF1 is associated with worse overall survival in GBM patients. MZF1 overexpression is proposed to contribute to the proliferation and growth of gliomas through the regulation of c-MYC [4]. Our findings suggest that MZF1 overexpression promotes the proliferation of patient-derived glioma cells. Using CTD and NCTD, we determined that they functioned as anti-glioma drugs, inhibiting the proliferation of gliomas. The results of our study demonstrate that CTD treatment is markedly more effective than NCTD treatment in inhibiting glioma proliferation and that CTD can effectively reduce the expression of MZF1. Taken together, these findings suggest that CTD may be useful for the treatment of glioblastoma by inhibiting the expression of MZF1.

## 2. Results

### 2.1. Overexpression of MZF1 Is Correlated with Poor Prognosis among Patients with GBM

**Figure 1:**
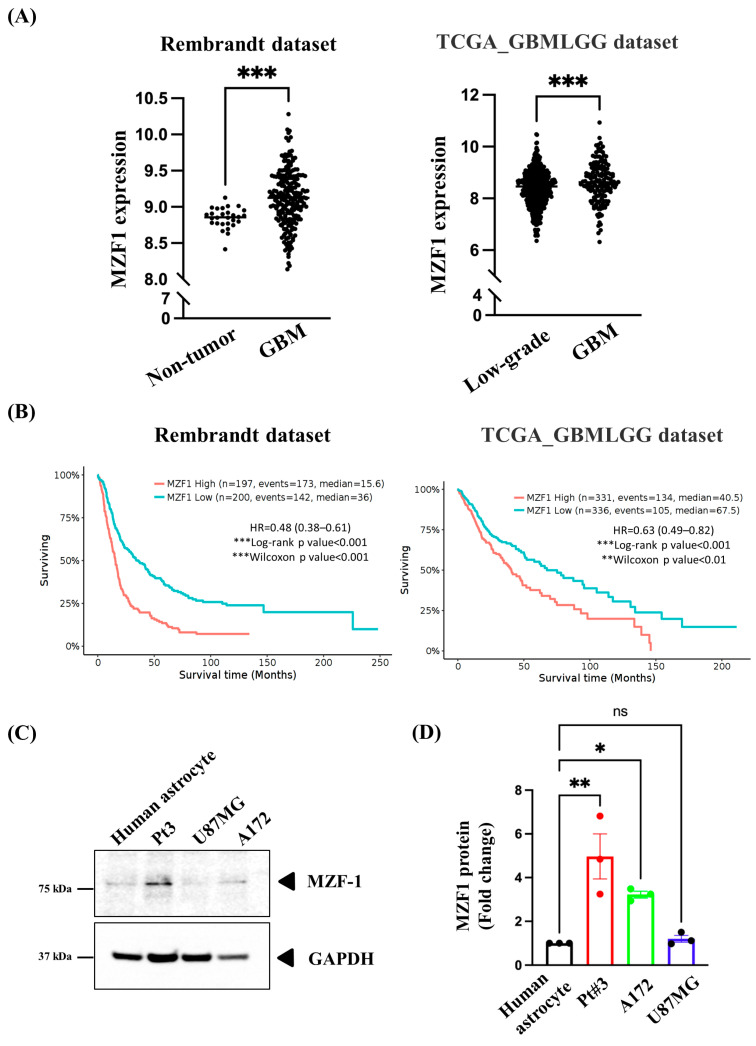
MZF1 was highly expressed in glioblastoma and associated with poor survival. Using the GlioVis data visualization tool (Rembrandt dataset and TCGA_GBMLGG dataset), we analyzed (**A**) MZF1 expression and (**B**) overall survival rates in GBM. MZF1 expression was demonstrated to be higher in GBM than in normal tissues and low-grade gliomas and correlated with a poorer prognosis. (**C**) Representative western blot image of the level of MZF1 protein in human primary astrocytes and gliomas (PT#3, A172, and U87MG). (**D**) Quantification of the data from (**C**) showed statistically significant increase in MZF1 levels in PT#3 and A172 cells compared with human primary astrocytes, but not in U87MG cells. Data are collected from three independent experiments and presented as mean ± SEM; one-way ANOVA followed by Dunnett’s multiple comparisons test, *p* = 0.0019 (human primary astrocytes vs. PT#3 cells) and *p* = 0.0435 (human primary astrocytes vs. A172 cells), * *p* < 0.05, ** *p* < 0.01, and *** *p* < 0.001. ns: no statistical significance.

Using bioinformatics analysis, we found that MZF1 expression was higher in glioma tissues than in non-tumor and low-grade glioma tissues (Figure 1A). Next, we examined whether high MZF1 expression is associated with patient survival. Figure 1B shows that patients with low MZF1 expression had better overall survival than those with high expression. MZF1 expression in glioma cells (PT#3 and A172) was significantly higher than that in primary astrocytes (Figure 1C,D). However, the expression of MZF1 was not significantly increased in U87MG cells. Therefore, our studies focused on PT#3 and A172 cells, which indicated that MZF1 may exert an oncogenic role in patients with GBM, whose expression correlates with poor overall survival.

### 2.2. MZF1 Overexpression Promotes Cell Proliferation and Regulates c-MYC Expression in PT#3 and A172 Glioma Cells

**Figure 2:**
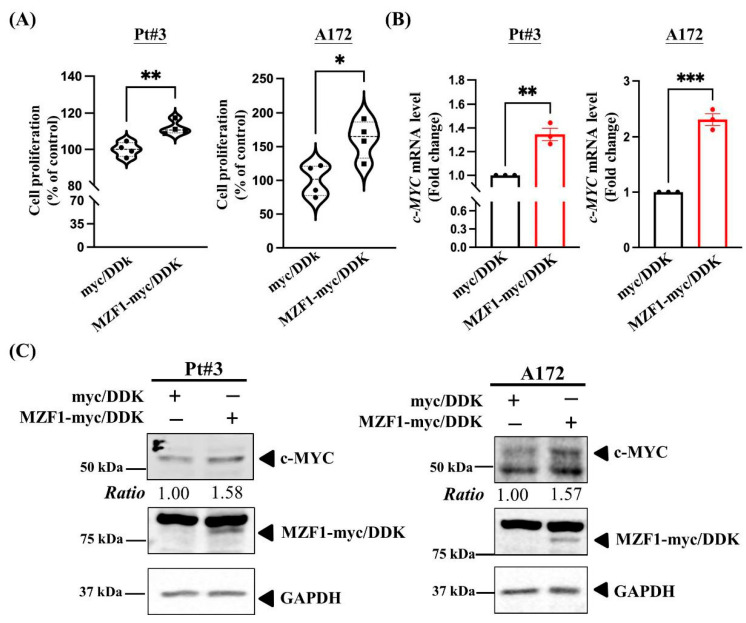
MZF1 overexpression promotes cell proliferation and increases c-MYC expression in PT#3 and A172 glioma cells. (**A**) A significant increase in cell proliferation was observed in MZF1-overexpressing PT#3 and A172 cells. Cell proliferation was assessed using the CCK8 assay, following the instructions provided with the kit. Data are collected from four independent experiments and presented as mean ± SEM; two-tailed unpaired Student’s *t*-test, *p* = 0.0048 (PT#3 cells); *p* = 0.0153 (A172 cells), * *p* < 0.05; ** *p* < 0.01. (**B**) The expression of *c-MYC* mRNA was increased in MZF1-overexpressing glioma cells. Real-time PCR analysis was conducted with c-MYC primers using cDNA harvested from MZF1-overexpressing PT#3 and A172 cells. Data are collected from three independent experiments and presented as mean ± SEM; two-tailed unpaired Student’s *t*-test, *p* = 0.0027 (Pt#3 cells) and *p* = 0.0002 (A172 cells), ** *p* < 0.01 and *** *p* < 0.001. (**C**) Western blot analysis demonstrated an increase in the c-MYC protein level in MZF1-overexpressing glioma cells. Cells transfected with empty vector were used as a control (myc-DDK).

MZF1 overexpression in different types of cancer reportedly promotes tumor progression [8,9], especially in glioma [4]. Therefore, we overexpressed MZF1 in gliomas to investigate cell proliferation. Using the CCK-8 assay, we determined whether the overexpression of MZF1 affected cell proliferation. Overexpression of MZF1 in PT#3 and A172 cells induced glioma proliferation after four and seven days, respectively (Figure 2A). Our result shows that the proliferation of glioma cells was enhanced by MZF1 overexpression. In addition, MZF1 overexpression regulated the transcription and translation of c-MYC (PT#3 and A172 cells; Figure 2B,C). These findings indicate that MZF1 overexpression was associated with regulating glioma cell proliferation.

### 2.3. Antiproliferative Effect of Cantharidin on Gliomas

**Figure 3:**
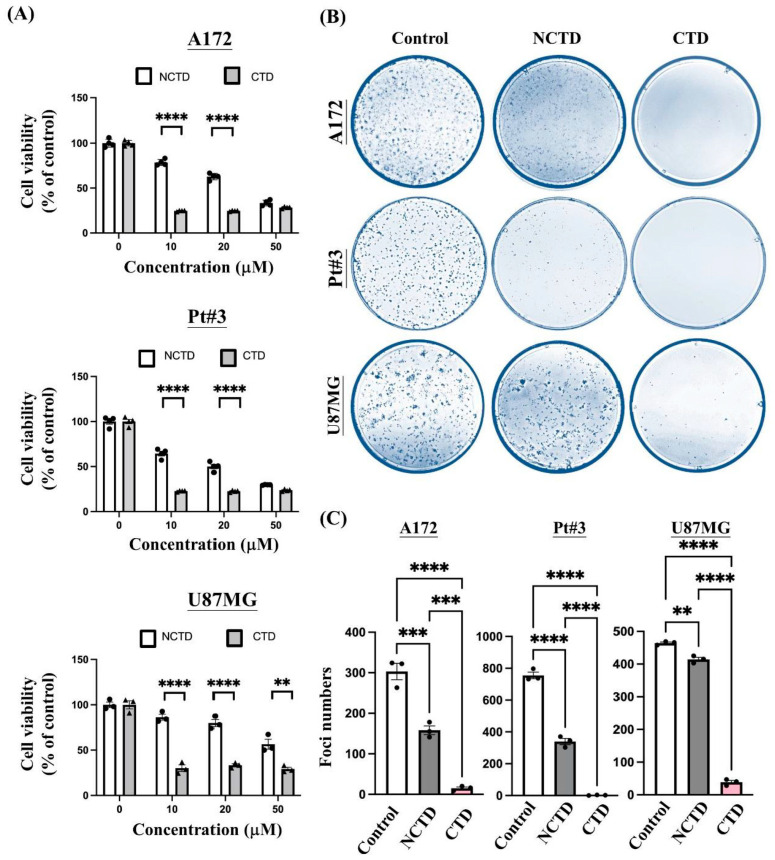
Cantharidin (CTD) had a greater inhibitory effect on glioma cell proliferation than norcantharidin (NCTD). (**A**) CTD exhibited a greater cytotoxic effect than NCTD in glioma cells (A172, PT#3, and U87MG). The cytotoxicity was evaluated using the CCK-8 assay. Data are collected from at least three independent experiments and presented as mean ± SEM; two-way ANOVA, ** *p* < 0.01 and **** *p* < 0.0001 (CTD vs. NCTD). The dose response analysis of CTD and NCTD in A172 and Pt#3 cells: 0 vs. 10, 0 vs. 20, and 0 vs. 50, *p* < 0.0001. The dose response analysis of CTD in U87MG cells: 0 vs. 10, 0 vs. 20, and 0 vs. 50, *p* < 0.0001; the effects of NCTD on U87MG cells: 0 vs. 20, *p* < 0.05; 0 vs. 50, *p* < 0.0001. (**B**) Crystal violet staining was used to examine colonies in A172, PT#3, and U87MG cells. (**C**) In contrast to the control group, data quantification of (**B**) revealed a decrease in colony numbers in both CTD and NCTD groups. Cells treated with the same concentration of DMSO were used as a control. Data are collected from four independent experiments and presented as mean ± SEM; one-way ANOVA followed by Tukey’s multiple comparisons test, *p* = 0.0006 (control vs. CTD) and *p* < 0.0001 (control vs. NCTD) in A172 cells; *p <* 0.0001 (control vs. CTD) and *p* < 0.0001 (control vs. NCTD) in PT#3 cells; *p <* 0.0012 (control vs. CTD) and *p* < 0.0001 (control vs. NCTD) in U87MG cells. ** *p* < 0.01, *** *p* < 0.001 and **** *p* < 0.0001.

The CCK-8 assay was used to validate the proliferation of glioma cells treated with high-dose CTD or NCTD. After 24 h of treatment, CTD significantly reduced the viability of glioma cells to a greater extent than NCTD (Figure 3A). Furthermore, we performed foci assays to determine how CTD and NCTD affect the long-term proliferation of glioma cells. CTD significantly inhibited colony formation in the foci assay compared with NCTD (Figure 3B,C).

**Figure 4:**
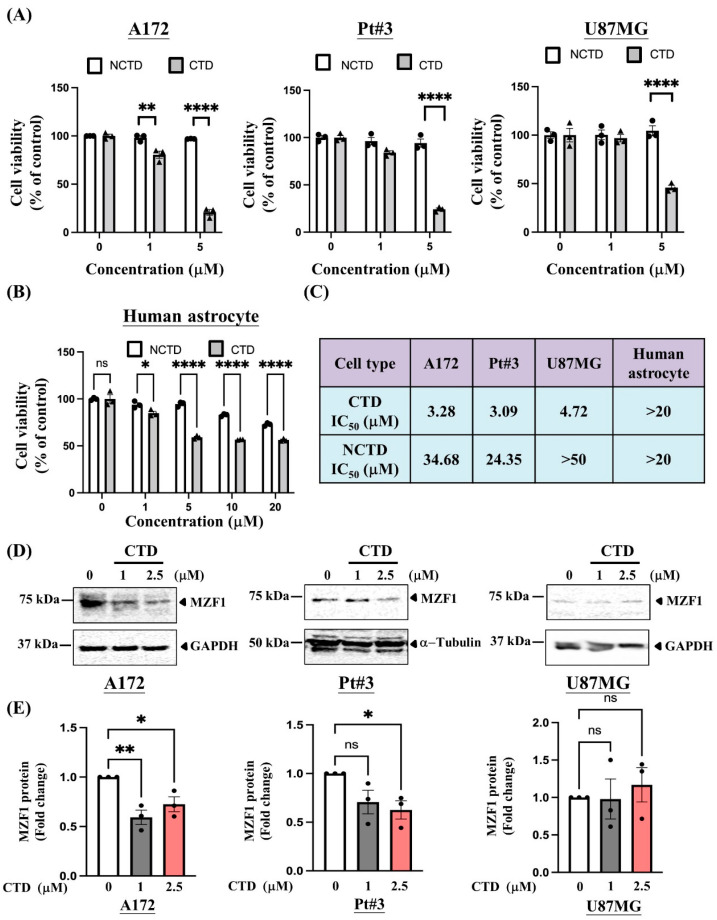
Cantharidin (CTD) reduces cell proliferation by inhibiting MZF1 expression. (**A**) Low-dose CTD significantly inhibited cell survival more than low-dose NCTD in glioma cells (A172, PT#3, and U87MG). The cell viability was determined using the CCK-8 assay. Data are collected from at least three independent experiments and presented as mean ± SEM; two-way ANOVA, ** *p* < 0.01 and **** *p* < 0.0001. The dose response analysis of CTD in A172 cells: 0 vs. 1, *p* < 0.001 and 0 vs. 5, *p* < 0.0001. The dose response analysis of CTD in Pt#3 cells: 0 vs. 1, *p* < 0.05 and 0 vs. 5, *p* < 0.0001. The dose response analysis of CTD in U87MG cells: 0 vs. 5, *p* < 0.0001. (**B**) Compared to glioma cells, primary astrocytes were resistant to the cytotoxicity caused by CTD. Data are collected from three independent experiments and presented as mean ± SEM; two-way ANOVA, * *p* < 0.05 and **** *p* < 0.0001. The dose response analysis of CTD in human astrocyte: 0 vs. 1, *p* < 0.001; 0 vs. 5, 0 vs. 10, and 0 vs. 20, *p* < 0.0001. The dose response analysis of NCTD in human astrocyte: 0 vs. 10 and 0 vs. 20, *p* < 0.0001. (**C**) The IC_50_ values of CTD and norcantharidin (NCTD) were determined using the CCK8 assay and a four-parameter logistic regression model in human primary astrocytes and glioma cells. (**D**) CTD treatment significantly decreased MZF1 protein levels in A172 and PT#3 cells, but not in U87MG cells. (**E**) A quantitative analysis of data collected in (**D**) revealed that MZF1 expression was significantly reduced in PT#3 and A172 cells after 24 h of CTD treatment. Cells treated with the same concentration of DMSO were used as a control. Data are collected from three independent experiments and presented as mean ± SEM; one-way ANOVA followed by Dunnett’s multiple comparisons test, * *p* < 0.05 and ** *p* < 0.01. ns: no statistical significance.

In addition, glioma cells were more susceptible to CTD treatment than human primary astrocytes (IC_50_ > 20 µM) (Figure 4A–C). These data indicate that CTD significantly reduces glioma proliferation compared with NCTD, and reveal less cytotoxicity in primary human astrocytes. Next, we examined whether CTD inhibited glioma proliferation by inhibiting MZF1 expression.

### 2.4. Cantharidin Disrupts MZF1-Induced c-MYC Expression in PT#3 and A172 Cells

**Figure 5:**
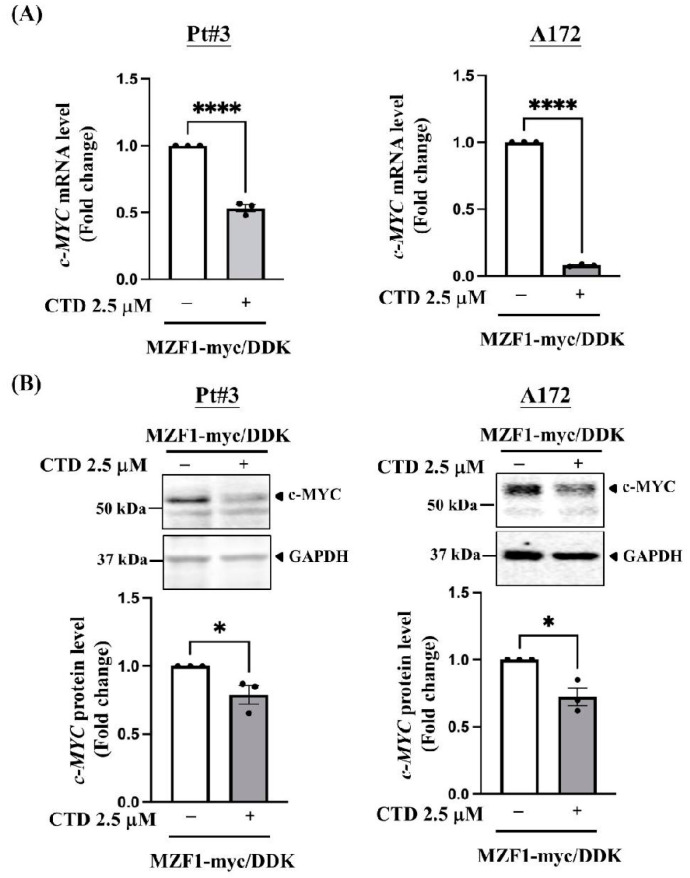
MZF1-induced expression of c-MYC was suppressed by cantharidin (CTD) in glioblastoma cells. (**A**) CTD treatment decreased the mRNA levels of c-MYC in MZF1-overexpressing glioma cells. Real-time PCR analysis was conducted with c-MYC primers using cDNA harvested from MZF1-overexpressing glioma cells. Data are collected from three independent experiments and presented as mean ± SEM; two-tailed unpaired Student’s *t*-test, **** *p* < 0.0001 (A172 cells, MZF1 overexpression vs. MZF1 overexpression + 2.5 μM CTD); **** *p* < 0.0001 (PT#3 cells, MZF1 overexpression vs. MZF1 overexpression + 2.5 μM CTD). (**B**) CTD treatment suppressed the protein levels of c-MYC in glioma cells. Cells treated with the same concentration of DMSO were used as a control. Data are collected from three independent experiments and presented as mean ± SEM; two-tailed unpaired Student’s *t*-test, * *p* < 0.0371 (PT#3 cells, MZF1 overexpression vs. MZF1 overexpression + 2.5 μM CTD); *p* < 0.0147 (A172 cells, MZF1 overexpression vs. MZF1 overexpression + 2.5 μM CTD).

We examined whether CTD treatment reduced MZF1 expression in glioma cells. After 24 h of treatment, CTD significantly decreased MZF1 protein expression in PT#3 and A172 cells, but not in U87MG cells (Figure 4D,E). We hypothesized that CTD suppresses c-MYC expression by downregulating MZF1 expression. We examined c-MYC expression in PT#3 and A172 cells that overexpressed MZF1. Interestingly, CTD treatment inhibited c-MYC expression at both the mRNA and protein levels in MZF1 overexpressing cells (Figure 5A and Figure 5B, respectively). Our combined data showed that CTD inhibited the MZF1/c-MYC axis in glioma cell proliferation.

## 3. Discussion

GBM displays abnormal proliferation and aggressive invasion of the surrounding normal brain tissue, resulting in an increased and irreversible recurrence following chemotherapy with temozolomide (TMZ) or surgery [5,18]. However, the exact mechanisms underlying glioma proliferation need to be elucidated. Although our study was conducted using cell culture models, the compelling results demonstrate the molecular mechanisms underlying glioblastoma progression. MZF1 is an oncogenic transcription factor that promotes cellular proliferation. To this end, we examined which Chinese medicines inhibited MZF1 expression in gliomas and prevented glioma cell proliferation.

MZF1 plays a key role in various cancers. According to our results, MZF1 plays an oncogenic role in promoting glioma cell proliferation. MZF1 is proven to be involved in oncogenic functions in other cancers, such as colorectal carcinoma [8], prostate cancer [19], lung adenocarcinoma [9], and hepatocellular carcinoma [20]. However, MZF1 has also been shown to play a tumor suppressor role in stomach adenocarcinoma [21]. The expression of miR-328-3p is directly activated by MZF1 in stomach adenocarcinomas, leading to reduced tumor growth [21]. MZF1 appears to serve different roles in different types of cancers. Based on the data collected in the present study, combined with bioinformatics analysis and cellular experiments, MZF1 was confirmed to act on a specific oncogenic pathway in gliomas. In this study, we found that the endogenous MZF1 protein levels in glioma cells (PT#3 and A172, but not U87MG) were markedly higher than that in primary astrocytes (Figure 1C,D). Additionally, we also found that CTD treatment (2.5 μM) did not affect the expression of MZF1 in U87MG cells (Figure 4D,E, right panels), whereas the MZF1 were suppressed in PT#3 and A172 cells under the same CTD treatment (Figure 4D,E, middle and left panels). The endogenous expression of MZF1 among different glioma cells may contribute to the genetic backgrounds, transcriptional and translational regulations, and posttranslational modifications. We speculate that MZF1 may not play a decisive role in the proliferation of U87MG cells.

CTD and its derivative NCTD are terpenoid compounds with molecular weights of 196.202 g/mol and 168.15 g/mol, respectively [22]. However, the ability of these drugs to penetrate the blood–brain barrier remains elusive. A previous study showed that a small molecular weight drug (<400 Da) may penetrate the blood–brain barrier [23]. In addition, NCTD can cross the blood–brain barrier, inhibiting medulloblastoma growth of medulloblastoma [10]. If this is the case, then CTD may be capable of crossing the blood–brain barrier. We also examined the effect of CTD, which inhibits MZF1, on the prevention of glioma proliferation. Nucleocytoplasmic transport has been reported to affect the translocation of MZF1 into the nucleus, inhibiting gene transcription in gliomas [4]. Approximately 30 nuclear nucleoporins (nuclear pore proteins) play an important role in nucleocytoplasmic transport [24]. Nevertheless, whether CTD inhibits nucleoporin expression to affect MZF1-regulated c-MYC transcription remains to be determined. CTD is reportedly a potent and selective inhibitor of protein phosphatase 2A (PP2A) that inhibits cell proliferation [25] and induces cell apoptosis [26]. Previous studies indicated that CTD increased the phosphorylation of GSK3β at Ser9 through inhibiting PP2A [27,28]. Moreover, GSK3β reduces MZF1 expression, leading to c-MYC downregulation in colorectal carcinoma [8]. Given the relationship between PP2A and GSK3β in regulating MZF1 and c-MYC expression, we speculate that CTD inhibits MZF1 expression via PP2A/GSK3β signaling pathway.

It is known that p53 status affects NCTD-induced apoptosis in glioblastoma cells [29]. Given the effect of NCTD on mutant p53 cells, cells with mutant p53 resisted NCTD-induced cytotoxicity. On the contrary, cells supplemented with wild-type p53 showed more sensitivity to NCTD-induced cytotoxicity. Our results showed that U87MG cells were significantly resistant to NCTD-induced apoptosis as compared with A172 cells (Figure 3B,C) (Note: A172 and U87MG are wild-type p53 glioma cells). These data pointed out the possibility of molecular mechanisms contributing to cell apoptosis under NCTD treatment.

Previous research demonstrated that long noncoding RNA 01060 (lncRNA01060) increases MZF1 translocation into the nucleus and promotes MZF1-regulated c-Myc, HK2, and PGK1 transcription in glioma, facilitating aerobic glycolysis and inducing tumor progression [4]. c-Myc reportedly contributes to the glioma cell proliferation via c-Myc-LPP-AS2 axis [30], promotes glioma stem cell formation via CDK8-regulated c-Myc expression [31], and enhances migration as well as angiogenesis via c-Myc-microRNA-9 axis [32]. More importantly, c-Myc exactly plays a critical role in tumorigenesis, in particular glioma progression. Here, we demonstrated that c-Myc expression was higher in glioma than in non-tumor tissue (Figure S1). These data are similar to that in Figure 1A—that MZF1 overexpression in GBM. Therefore, MZF1 and c-Myc indeed contribute to glioma tumorigenesis.

As the treatment of GBM demands potent delivery of anti-cancer drugs to the brain, developing a more specific delivery approach for bulk glioma treatment is essential. Exosomes are being used as drug carriers in anti-cancer treatment and are regarded as safe and effective carriers. Several therapeutic agents can be loaded and delivered by exosomes, including small-molecule drugs, mRNA, miRNAs, and protein [33]. Exosomes can be utilized as chemotherapeutic drugs and microRNA carriers, such as the exosome-based formulation of paclitaxel to inhibit multiple drug-resistant (MDR) cancer cells [34] and exosome-derived microRNA-146 to inhibit glioma growth [35]. Therefore, CTD-loaded exosomes and conjugate-specific glioma surface markers can be used in the future to target glioma bulk through nasal or intravenous injection.

In conclusion, MZF1, an oncogenic transcription factor, promotes glioma proliferation by inducing c-MYC expression. Our data suggest that CTD treatment diminishes glioma proliferation, which is associated with the down-regulation of MZF1 and c-MYC. We anticipate that CTD could serve as a therapeutic agent for the treatment of gliomas in the future.

## 4. Materials and Methods

### 4.1. Cell Culture and Transfection

Human glioma cell lines A172 and U87MG were purchased from the American Type Culture Collection (ATCC, Manassas, VA, USA). PT#3 glioblastoma cells were kindly provided by Dr. Jian-Ying Chuang at Taipei Medical University. The cells were cultured and maintained in Dulbecco’s modified Eagle’s medium (DMEM, GIBCO, Waltham, MA, USA ) containing 10% fetal bovine serum (FBS, CORNING, REF 35-010-CV, Glendale, AZ, USA) and 1% penicillin/streptomycin (GIBCO, Waltham, MA, USA). Human primary astrocytes were purchased from ScienCell^TM^ Research Laboratories (Catalog #1800, Carlsbad, CA, USA) and grown in the astrocyte medium (Catalog #1801) with 10% fetal bovine serum (FBS, Cat. No. 0010), and 5 mL of an astrocyte growth supplement (AGS, Cat. No. 1852). Myc-DDK tagged MZF1 expression plasmid and control plasmid were purchased from Origene (Catalog RC220791 and PS100001, respectively) (Rockville, MD, USA). Cells at 70% confluency were transfected with plasmids using PolyJet reagent (SignaGen Laboratories, Gaithersburg, MD, USA). The transfection reagent and plasmids were mixed at a ratio of 2:1 and then incubated for 20 min at room temperature in serum-free medium (0.5 mL). Subsequently, the mixture was added to a culture dish and then incubated at 37 °C in a 5% CO2 incubator (Thermo Fisher Scientific, Waltham, MA, USA) for 24 h.

### 4.2. Bioinformatic Analysis

A GlioVis data visualization tool was used to perform gene comparisons (http://gliovis.bioinfo.cnio.es. The data was accessed on 15 June 2022). Additionally, the overall survival rates for glioblastoma patients were analyzed using the same bioinformatic tools. The datasets used in this study are the Rembrandt dataset and the TCGA_GBMLGG dataset.

### 4.3. CCK-8 Assay

The experimental procedures were performed according to the instructions of the CCK-8 assay. Briefly, the cells were seeded and incubated in 24-well plates at 37 °C overnight. CTD (MERCK, C7632, Kenilworth, NJ, USA) and NCTD (MERCK, N8784) were dissolved in DMSO (MERCK, D8418). Subsequently, cells were treated with CTD or NCTD for 24 h. Cells treated with the same concentration of DMSO were used as a control. After 24 h, the treated cells were incubated with CCK-8 reagent for 1 h at 37 °C, and the signals were detected at 450 nm using a SpectraMax^®^ iD3 reader. The IC_50_ (inhibitory concentration of half maximum) values of CTD and norcantharidin (NCTD) were determined by using a four-parameter logistic regression model (Prism software version 9.4.0, San Diego, CA, USA).

### 4.4. Foci Assay

Cells were seeded in 60-mm dishes (2000 cells/dish) and treated with CTD or NCTD for 24 h. After incubation, the culture media were changed and the cells were incubated at 37 °C for another 7 d. After incubation, the colonies were stained with 0.05% crystal violet at room temperature for 16 h, and the signals were detected using an Azure Biosystem 400 Imaging System. The stained cells were analyzed using AzureSpot Pro software (Dublin, CA, USA).

### 4.5. Western Blot Analysis

The cells were harvested and lysed in an immunoprecipitation lysis buffer (50 mM NaCl, 0.5% Nonidet P-40, and 10 mM Tris-HCl, pH 8.0) containing EDTA-free protease inhibitor cocktail tablets (Complete Mini, EDTA-free) on ice for 30 min. Further, equal concentrations of proteins were denatured with sodium dodecyl sulfate (SDS) 4X sample buffer (Bio-Rad, Hercules, CA, USA) containing 1% 2-ME (2-mercaptoethanol) and heated at 95 °C for 10 min. These samples were separated using 10% SDS-polyacrylamide gel electrophoresis (SDS-PAGE) and transferred onto polyvinylidene difluoride (PVDF) membranes. After incubation with 5% nonfat milk in TBST (Tris-buffered saline with 0.1% Tween 20) for 1 h, membranes were incubated with primary antibodies overnight at 4 °C (Table S1). The membranes were washed three times with TBST for 10 min, followed by incubation with secondary antibodies for 1 h at room temperature. Blots were washed three times for 10 min with TBST and developed using the Azure Biosystem 400, and the band intensity was analyzed using the Image Studio Lite software (LiCor 5.2.5, Lincoln, NE, USA).

### 4.6. RT-qPCR for Quantifying Gene Expression

Total RNA was extracted from treated and untreated cells following the manufacturer’s instructions (RNeasy Mini Kit; Qiagen, Hilden, Düsseldorf, Germany). Reverse transcription was performed using the iScript cDNA Synthesis kit (Bio-Rad). Subsequently, quantitative PCR was conducted using the iTaq Universal SYBR Green SuperMix kit (Bio-Rad) with specific primers and cDNA to quantify gene expression using the 2^−ΔΔCt^ method. The sequences of specific primers were as follows: MZF1 (F) 5′-TCCAGGTAGTGTAAGCCCTCA-3′; MZF1 (R) 5′-TCCTGTTCACTCCTCAGATCG-3′; c-MYC (F) 5′- GTCAAGAGGCGAACACACAAC-3′; c-MYC (R) 5′-TTGGACGGACAGGATGTATGC-3′; GAPDH (F) 5′-GAGTCAA CGGATTTGGTCGT-3′; and GAPDH (R) 5′-GACAAGCTTCCCGTTCTCAG-3′.

### 4.7. Statistical Analysis

Data were collected from at least three independent experiments and compared for statistical significance using the Prism software (version 9.4.0). Data were collected from experiments performed in replicates and expressed as the mean ± SEM. Unpaired Student’s *t*-test and one-way or two-way analysis of variance (ANOVA), followed by the appropriate multiple comparisons test, were used to investigate statistical significance. * *p* < 0.05, ** *p* < 0.01, *** *p* < 0.001, and **** *p* < 0.0001 indicate statistical significance.

## Data Availability

All data generated during this study are included in this article.

## Supplementary Materials

The following supporting information can be downloaded at: https://www.mdpi.com/article/10.3390/ijms232314727/s1.
