# Supplementary material for: Inhibition of MZF1/c-MYC Axis by Cantharidin Impairs Cell Proliferation in Glioblastoma
Source: Int J Mol Sci. 2022 Nov 25;23(23):14727. doi: 10.3390/ijms232314727 (PMC9740304; doi:10.3390/ijms232314727)
Supplement: Supplementary file 1 [file ijms-23-14727-s001.zip › ijms-2028029-supplementary.pdf]

Table S1. Information of antibodies used in this study.

Antibodies	Species	dilution ratio; WB*	Source	Category No.
MZF1	mouse	WB: 1:1000x	Santa Cruz Biotechnology	sc-293218
GAPDH	mouse	WB: 1:10000x	EMD Millipore	MAB374
c-MYC	rabbit	WB: 1:1000x	Abcam	ab32072
DDK (Flag)	rabbit	WB: 1:1000x	Cell Signaling Technology	#14793
alpha-Tubulin	mouse	WB: 1:10000x	Proteintech Group, Inc.	66,009-1-lg

*WB: western blot

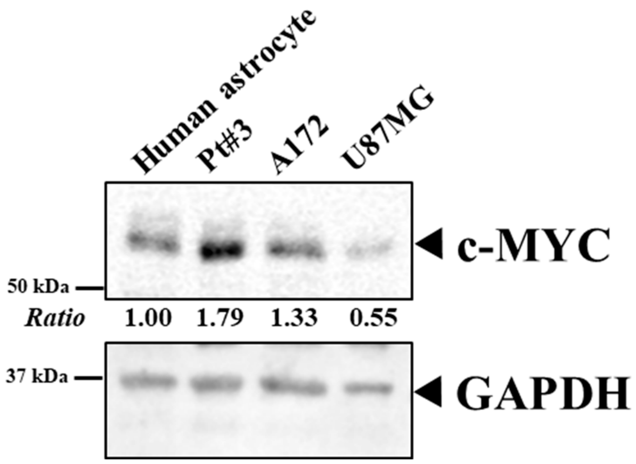

Figure S1. The endogenous c-MYC protein levels in Pt#3 and A172 cells are markedly higher than those in human astrocyte and U87MG cells. Western blot image of the level of c-MYC protein in human primary astrocytes and gliomas (PT#3, A172, and U87MG).
